# Comparative Molecular and Metabolic Profiling of Two Contrasting Wheat Cultivars under Drought Stress

**DOI:** 10.3390/ijms222413287

**Published:** 2021-12-10

**Authors:** Hind Emad Fadoul, Félix Juan Martínez Rivas, Kerstin Neumann, Salma Balazadeh, Alisdair R. Fernie, Saleh Alseekh

**Affiliations:** 1 Department of Botany, Faculty of Science, University of Khartoum, Khartoum 11115, Sudan; 2 Department of Biology, University of Toronto Mississauga, Mississauga, ON L5L 1C6, Canada; 3 Max Planck Institute of Molecular Plant Physiology, Am Mühlenberg 1, 14476 Potsdam-Golm, Germany; rivas@mpimp-golm.mpg.de (F.J.M.R.); balazadeh@mpimp-golm.mpg.de (S.B.); fernie@mpimp-golm.mpg.de (A.R.F.); 4 Leibniz Institute of Plant Genetics and Crop Plant Research (IPK), Corrensstrasse 3, 06466 Seeland, Germany; neumannk@ipk-gatersleben.de; 5 Institute of Biology, Leiden University, Sylviusweg 72, 2333 BE Leiden, The Netherlands; 6 Center of Plant Systems Biology and Biotechnology, Ruski Blvd. 139, 4000 Plovdiv, Bulgaria

**Keywords:** wheat, drought tolerance, JUNGBRUNNEN1 (JUB1), metabolomics, lipids

## Abstract

Drought is one of the most important threats to plants and agriculture; therefore, understanding of the mechanisms of drought tolerance is crucial for breeding of new tolerant varieties. Here, we assessed the effects of a long-term water deficit stress simulated on a precision phenotyping system on some morphological criteria and metabolite traits, as well as the expression of drought associated transcriptional factors of two contrasting drought-responsive African wheat cultivars, Condor and Wadielniel. The current study showed that under drought stress Wadielniel exhibits significant higher tillering and height compared to Condor. Further, we used gas chromatography and ultra-high performance liquid chromatography mass-spectrometry to identify compounds that change between the two cultivars upon drought. Partial least square discriminant analysis (PLS-DA) revealed that 50 metabolites with a possible role in drought stress regulation were significantly changed in both cultivars under water deficit stress. These metabolites included several amino acids, most notably proline, some organic acids, and lipid classes PC 36:3 and TAG 56:9, which were significantly altered under drought stress. Here, the results discussed in the context of understanding the mechanisms involved in the drought response of wheat cultivars, as the phenotype parameters, metabolite content and expression of drought associated transcriptional factors could also be used for potential crop improvement under drought stress.

## 1. Introduction

Wheat (*Triticum aestivum* L.) is the most cultivated crop worldwide in terms of cultivated area and grain acreage (FAO, 2020), and accounts for 20% of the carbohydrate and protein sources in the human diet [1]. In the context of global climate change, crop tolerance to abiotic stresses such as drought, heat, salt, water logging, and freezing is becoming more critical to ensure food access worldwide. Environmental stresses and climatic extremes represent the main constraints of crop productivity. Wheat production is highly affected by environmental stresses; changes in weather conditions are affecting crops in Europe and in the Indian subcontinent, where the increase in the temperature is reducing wheat yield up to 0.2–0.5 t ha^−1^ [2,3]. Moreover, up to 40% of the inter-annual wheat yield variability in the period 1980–2010 is attributed to extreme weather changes, such as heat waves and periods of drought [4].

Among the abiotic stresses that affect plants, drought is one of the main factors which reduces leaf and root growth, as well as yield [5]. With an estimated third of the total cultivated area affected by drought stresses, improving the knowledge related to the molecular mechanisms underlying drought tolerance is a critical factor needed for the development of new drought tolerant genotypes [6,7]. Drought tolerance includes a wide range of genetic, biochemical, and physiological adaptations displayed by plants to cope with this stress. One of the responses is to synthetize new proteins that help to maintain homeostasis and water potential in the cell. This change in the transcriptome is driven by the expression of transcription factors (TFs), which regulate the expression of some stress resistant genes [8]. Different TF families have been related with the regulation of drought responses in wheat such as NAM, ATAF, and CUC (NAC), dehydration-responsive element-binding (DREB) factors, basic leucine zipper (bZIP), MYB, and WRKY [9].

Another response used by plants is to reconfigure its metabolic networks to acclimate to the stress condition. Thus, metabolic studies have been performed to decipher how plants adapt to new conditions. Higher levels of some metabolites, such as proline, different sugars, or polyols are increased not only in drought, but also in response to other abiotic stresses, revealing that plants present a core metabolic response to face different stresses [10]. In wheat, higher levels of metabolites, such as tricarboxylic acid intermediates, sugars, and amino acids, have been described in response to drought [11,12]. Moreover, lipids also respond to stresses in plants, as these conditions induce fatty acid saturation, thus increase the rigidity of the membrane. In addition, reactive oxidative species (ROS), produced due to stress conditions, will cause oxidative damage to the membrane, causing a malfunction in cell membranes [13,14]. Cell membranes are of the first receptors of drought stress, and they can protect the cell by changing stress perception and rigidity of the cell structure. For example, changes in membrane lipid such as phosphate dylcholine (PC), phoshatidylethanolamine (PE), digalactosyldiacylglycerol (DGDG), and monogalactosyldiacylglycerol (MGDG) may compromise the response against drought [15]. On the other hand, under water stress, a decrease in membrane lipid content was observed, and PC was largely degraded [16].

In this work, we have studied the different drought response of two wheat cultivars contrasting in their drought tolerance and extensively used by African farmers, including physiological responses and the expression patterns of TFs related to drought, as well as metabolic profiling. The information provided will improve our knowledge concerning the drought response in wheat that contributes to a better understanding of the basis of plant stress responses.

## 2. Results

### 2.1. Phenotypic Alteration on Water Limitation Stress

**Figure 1:**
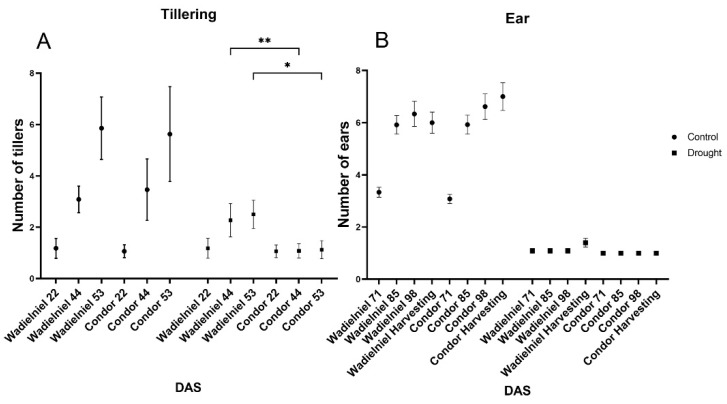
Phenotypical traits in wheat cultivars in response to water deficit stress. (**A**). Number of tillers observed and (**B**) number of ears in both cultivars. Samples were taken in different timepoints DAS 22, 44, and 53 for tillering and DAS 71, 85, 98, and at harvesting day for ear number. Ear number was inferred from visual inspection of images taken by the phenotyping system. Bars show mean ± SD. Statistical significances are determined by a Student *t*-test between cultivars in the same experimental conditions * *p* < 0.05, ** *p* < 0.01.

Regarding tiller numbers, both cultivars presented a similar pattern with no differences in control treatment, and plants formed in average one tiller at DAS 22 (day after sowing 22, T0) to five tiller at DAS53 (Figure 1A). After the water deficit treatment started, Condor (the drought sensitive cultivar) stopped tillering, showing no further increase from T0 until the end, with around one tiller across the whole treatment. Meanwhile, Wadielniel (the drought tolerant cultivar) continued to form tillers after T0, up to three at DAS53 (Figure 1A). After flowering, ear formation did not show any differences between the two cultivars in the evaluated period, with both cultivars producing around one per plant in drought. Still, Wadielniel presented a slightly higher number of ears under the stress condition due to late formation of secondary ears, but this was not statistically significant (1 vs. 1.4 on average for Condor and Wadielniel, respectively). However, these secondary ears were sterile and produced no seeds. No differences in ear number were observed in the control treatment (Figure 1B).

**Figure 2:**
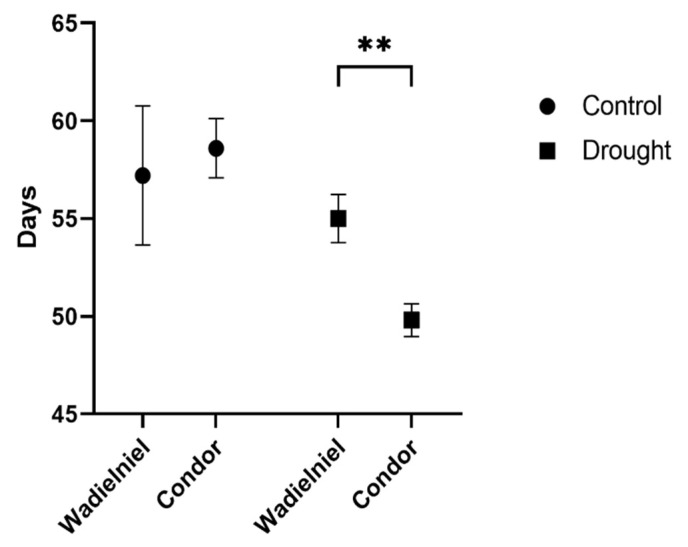
Days of heading for both cultivars in control and water limitation condition as inferred from pictures. Bars show mean ± SD. Statistical significances are determined by a Student *t* test between cultivars in the same experimental conditions ** *p* < 0.01.

The time of heading, BBCH55 [17] was inferred from inspecting the images. Under control conditions, no difference was seen with this treatment, as both cultivars presented a heading time of around 57 days (Figure 2). Both cultivars responded to water deficit stress by earlier heading compared to the control, with this being more pronounced in Condor, which headed, on average, three days earlier than cv. Wadielniel (Figure 2). This difference indicates that Condor reacts more sensitively to drought, using early heading as a stress escape strategy.

**Figure 3:**
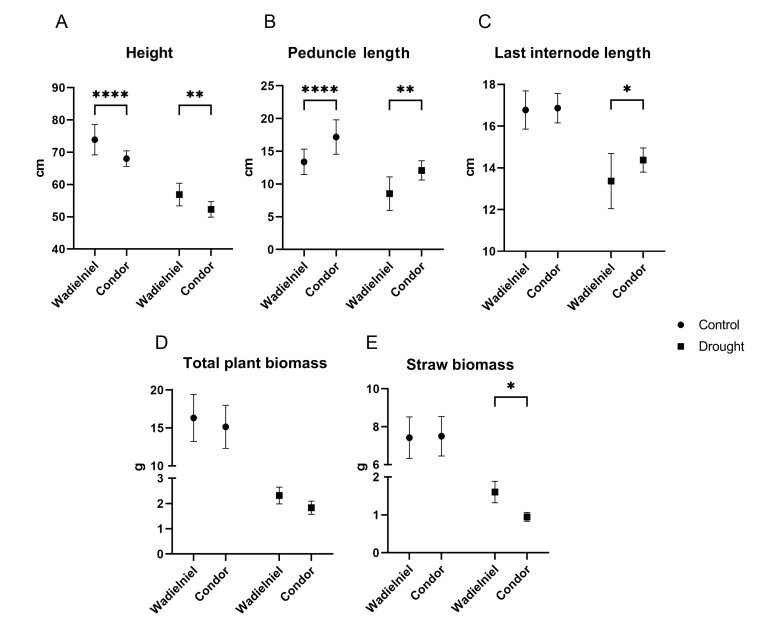
Different phenotypic parameters measured in both cultivars in response to water limitation. (**A**). Total plant height in cm, (**B**). Peduncle length in cm, (**C**). Last internode length, (**D**). Total plant biomass as sum of straw and grain biomass in grams, (**E**). Straw biomass in grams. Bars show mean ± SD. Statistical significances are determined by a Student *t*-test between cultivars in the same experimental conditions * *p* < 0.05, ** *p* < 0.01, **** *p* < 0.0001.

The drought tolerant cultivar Wadielniel was significantly taller in both treatments, with a difference of approximately 5 cm (Figure 3A). In contrast, Condor had a longer peduncle and awn length in both control and water deficit, as well as a longer last internode (Figure 3B,C). Wadielniel had a tendency of maintaining more biomass under stress (Figure 3D, *p* = 0.07) than Condor. Plant straw weight was significantly higher under water deficit for Wadielniel, due to the higher tillering (Figure 3E).

While Wadielniel developed more spikelets per ear than Condor under both control and drought conditions, Condor was able to produce more seeds per spikelet in both treatments (Table 1). As a consequence, plant grain number and grain number of the main ear did not significantly differ in the two cultivars, although there was a tendency for a higher number of grains in the main ear for Condor (*p* = 0.08) in control (Table 1).

The thousand-kernel weight (TKW) of the main ear and the average seed area were significantly higher in Wadielniel both under control and drought conditions, as well as seed width and length (Table 2).

In summary, Wadielniel does not clearly emerge as the better genotype at maturity on a single plant basis (no difference in grain yield), but some grain yield components were increased as compared to Condor, such as higher ear number, higher straw weight, and a trend of higher total biomass at maturity. The drought tolerant cultivar showed a higher tillering capacity under drought and did not react with drastic earlier heading as was seen for Condor.

### 2.2. Relative Expression of Drought Related Genes

**Figure 4:**
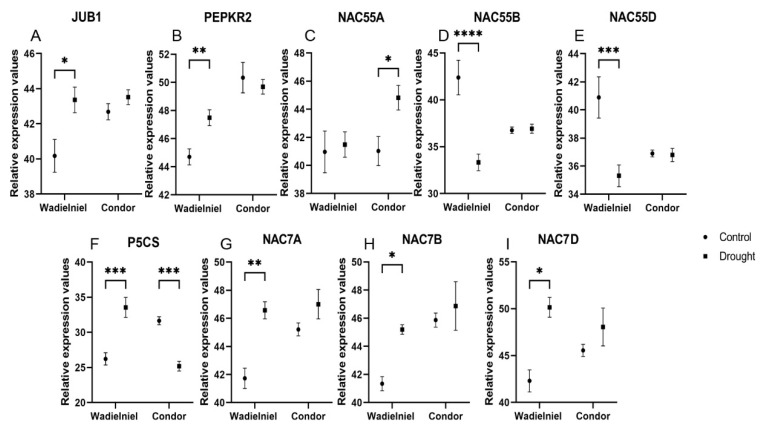
Relative expression data for selected genes studied by qRT-PCR. (**A**–**I**) Wheat genes selected for expression analysis. The primers used are listed in Table S2. The Y axis indicates expression level (40-ΔCt). Values are expressed between an arbitrary value of 40 and ΔCT, so a high 40-ΔCt value indicates a higher expression level. Statistical significances were determined with a Student *t*-test versus the control in the same cultivar. * *p* < 0.05, ** *p* < 0.01, *** *p* < 0.001, **** *p* < 0.0001.

As part of the plant response to drought, the expression of several TFs is modulated to cope with the adverse conditions. In that sense, we studied the expression of TFs related with drought response. TFs such as the *TaJUB1/NAC042* orthologue of *Arabidopsis thaliana ANAC042,* as well as *PEPKR2,* which encodes a serine/threonine protein kinase, increased expression in response to drought in the tolerant cultivar Wadielniel, while no change was observed in Condor (Figure 4A,B). We measured the three copies of *NAC55* found in the three chromosomes of wheat. While *NAC55A* increased expression in Condor, *NAC55B* and *NAC55D* were decreased in Wadielniel in response to water deficit stress (Figure 4C–E). Moreover, the expression of the delta-1-pyrroline-5-carboxylate synthase (*P5CS*) gene involved in proline biosynthesis, as a well-known osmolyte was upregulated in Wadielniel, but downregulated in Condor (Figure 4F), while in contrast to *NAC55*, all three copies of *NAC7* increased their expression in Wadielniel (Figure 4G–I).

### 2.3. Metabolite Profiling under Drought Stress

**Figure 5:**
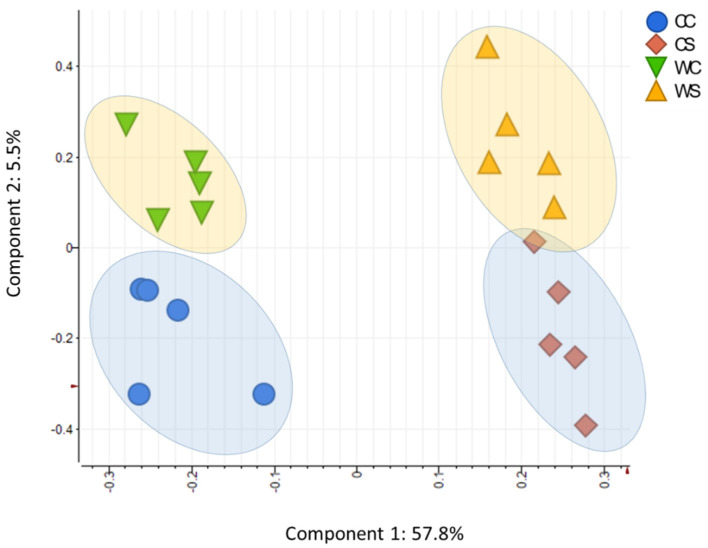
Partial least squares discriminant analysis (PLS-DA) based on both primary metabolites and lipids. CC, Condor Control; CS, Condor Stress; WC, Wadielniel Control; WS, Wadielniel Stress. Data from five independent biological replicates were used to perform the PLS-DA.

To explore the response upon drought in the two wheat cultivars, we used gas chromatography–mass spectrometry (GC-MS) and ultra-high performance liquid chromatography–mass spectrometry (UPLC-MS) to determine the levels of >150 compounds in leaf tissue. We were able to identify and quantify a total of 74 metabolites, including amino acids (28%), organic acids (23%), sugars (10%), polyamines, and nucleobases. For lipids, we were able to determine up to 78 different compounds. Among these highlights, 26 triacylglycerides TAGs (33% of the total) and 16 phosphatidylcholines PCs (20% of the total), the two main categories, were identified. To identify metabolites associated with the drought condition, principal component analysis (PCA) was performed (Figure 5). The first and the second components explained 57.8% and 5.5% of the metabolite variance, respectively.

**Figure 6:**
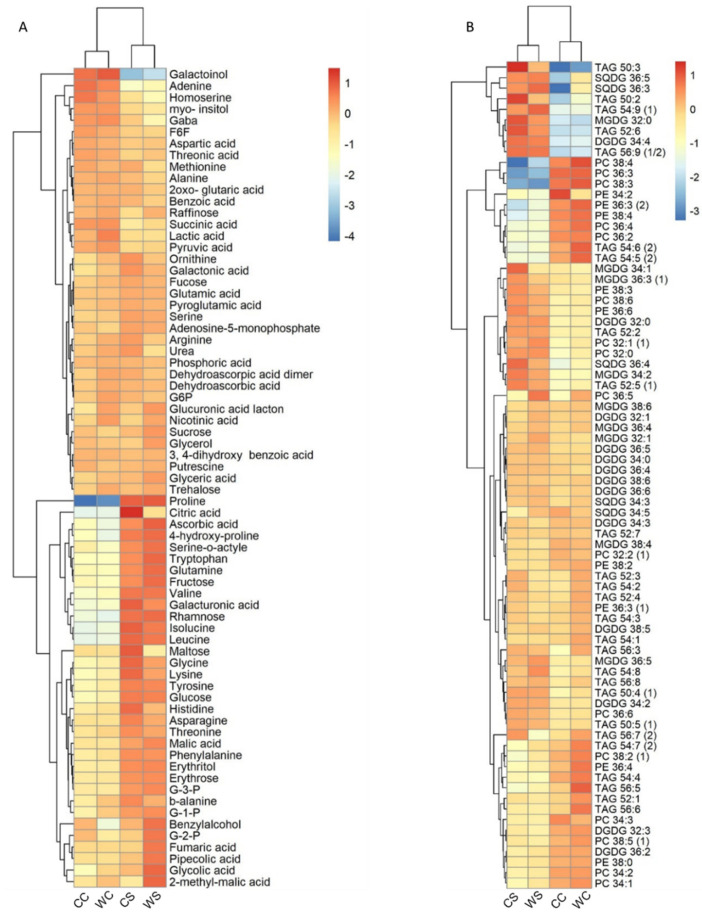
Heat map of primary metabolites measured by GC-MS (**A**) and lipids measured by UPLC-MS (**B**). Values correspond to *log2*-fold changes in relation to control samples of the same genotype. Full data sets are provided in Table S1.

**Figure 7:**
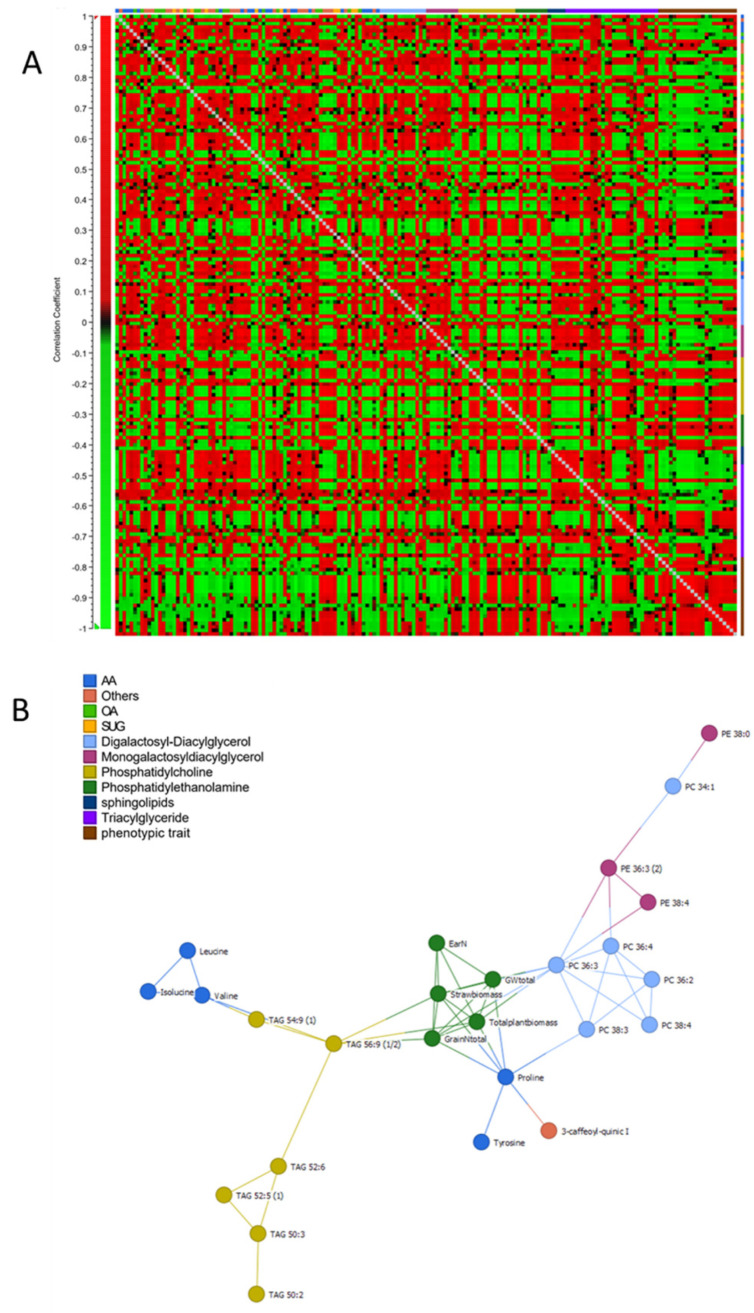
(**A**) Correlation analysis between and within phenotypic and metabolic traits. (**B**) Plan showing correlation network analysis with ≥90%, each node represents a metabolite or a plant phenotypic trait, edges connecting two nodes show an association between two traits. Full data represent in Table S3.

The results demonstrated that under normal conditions, no significant differences between the two cultivars were observed, as shown in the PCA (Figure 5). On the other hand, a total of 42 metabolites in Condor and 26 in Wadielniel were significantly changed (*p* < 0.05) under drought conditions (Table S1). Some metabolites exhibited a similar change in response to drought stress in both cultivars, i.e., a significant increase in the concentrations of free amino acids (proline, glycine, isoleucine, leucine, phenylalanine, tyrosine, valine, serine-o-acetyle, and glutamine), organic acids (glycolic acid, galacturonic acid and citric acid), and other compounds (G-1-P, G-3-P, erythrose, erythritol) were observed (Figure 6, Table S1). Meanwhile, a significant decrease of quinic acid, 3-caffeoyl-quinic acid, *myo*-inositol, and homo-serine were also observed. The metabolites that changed most significantly upon drought were proline with a 5-fold change and quinic acid (-7.4-fold change). For lipids, 35 in Condor and 33 Wadielniel were significantly changed in response to drought (Table S1). Among them, we observed a general higher concentration of TAGs in both cultivars, especially TAG 50:3 and TAG 54:9, but a lower concentration of PCs, such as 38:3 and 38:4 (Figure S1). Finally, we investigated correlations between and within different metabolite classes and phenotypic traits (Figure 7A, and Table S3). This revealed that many metabolites are highly associated within the network. Interestingly, strong correlations (>90%) were observed between some of the phenotypic traits and metabolites content (Figure 7B). For example, the amino acid proline was negatively correlated with total plant biomass, grain number, ear number, and straw biomass. In addition, the lipid classes PC 36:3 and TAG 56:9 were correlated with total plant biomass, straw biomass and total grain number (Figure 7B). This indicates that these compounds could be used as markers to assess the response to drought in wheat plants.

## 3. Discussion

Knowledge concerning the mechanisms underlying drought tolerance is important for the development of drought tolerant wheat genotypes. In this study, phenological, transcriptional, and metabolic responses of two wheat cultivars with contrasting drought stress responses were investigated under control and drought stress conditions.

### 3.1. Measures of Growth and Yield Related Traits in Response to Drought

The impact of drought on plant development depends on the timing of its occurrence. In the tillering phase, the number of tillers per plant and biomass is reduced [18]; during stem elongation, and the plant height is affected [5]; when water deficit stress occurs around the flowering stage, the seed set is negatively affected [19], while drought during the grain filling phase reduces the TKW [20,21]. In our experiments, the stress started in the early tillering stage, and lasted until seed maturity. Accordingly, all of these traits were reduced in drought compared to control treatment. While Wadieniel could continue tillering, albeit less than in control, Condor completely stopped tillering due to the ongoing drought. On the one hand, Duggan et al. (2000) showed that under drought stress, only small differences in grain yield were observed, while high kernel number, through greater tillering, was shown to be an adaptation to low-stress conditions [22]. Others indicated that some drought tolerant cultivars (e.g., Excalibur) found to be more responsive to cyclic drought stress, produced more tillers per se and aborted them under drought stress, and showed rapid recovery after re-watering [23].

Moreover, water deficit stress caused an earlier flowering in both cultivars, but this was much stronger in the sensitive cultivar Condor. However, in the control treatment, there was no statistical difference in flowering time. This indicates that Condor reacts more sensitive to stress and trying to escape from it by a faster development. It has been shown that early flowering and maturity are effective drought escape mechanisms in plants including cereal crops such as wheat [24]. It is argued that plants under water limitation condition attempt to survive by completing their life cycle quickly [25], and the genotypes sensitive to stress start to respond to drought at germination and tillering stage by producing less plants and aborting initial tillers. Khan et al. (2012) proposed that the reason behind reduced growth and early flowering under severe heat stress could be attributed to limitations of resources and the sensitivity of metabolic processes that have limit the ability to effectively utilize available resources [26].

In conclusion, the drought tolerant cultivar did show improved performance under drought conditions, compared with the sensitive cultivar, also under controlled greenhouse conditions in a pot experiment on a phenotyping platform. However, the yield advantage seen in the field was not achieved in this pot study. It might be that the stress conditions were too harsh to well differentiate the genotypes in their yield response. The threshold of 20% PAW (plant available water) was chosen based on the results of screening a diverse barley collection on the very same platform [18]. The stop of growth in barley occurred in average at 21% PAW. However, in this older setup, plants were re-watered after a ~3 weeks drought period. In our current experiment, wheat plants in drought treatment were grown at this water level up to maturity for the first time. Adjustment of the drought stress level lasting until maturity might yield better differentiation of contrasting genotypes in future studies on the platform.

### 3.2. Drought Affects Gene Expression

Signaling pathways of drought stress in plants involve several molecules, i.e., TFs, enzymes, functional proteins, molecular chaperones, and metabolites [27]. One of the most responsive elements are transcription factors (TF), which are key regulatory proteins, and play their role in activating or repressing gene expression and regulating many biological processes. Many TF genes are affected by drought, including members of the NAC family, dehydration-responsive element-binding (DREB) factors.

Here, we analyzed the expression of selected drought-associated TFs in the two wheat cultivars under drought stress condition. Wheat orthologues of the Arabidopsis gene *JUB1/ANAC042*, as well as *TaNAC7* transcripts of the three chromosome copies A, B, and D showed increased expression in the drought tolerant cultivar, but no significant changes in the sensitive cultivar Condor. Meanwhile, related to *NAC55*, only *NAC55A* increased its expression in Condor, while *NAC55B* and *D* were repressed in Wadielniel. Induction of the expression of NAC TFs upon abiotic stresses was reported in different plant species [28,29,30,31]. For example, Thirumalaikumar et al. (2018) showed that expression of *SlJUB1* in tomato is strongly induced upon treatment with H_2_O_2_, NaCl, PEG, and dehydration, indicating a role for this TF in the regulation of abiotic stress response networks in this plant, and concluded that JUB1 is a positive regulator of the response to drought in tomato [30].

In our study, the drought tolerant wheat cultivar Wadielniel exhibited an upregulation of *TaPEPKR2,* while no change was recorded for the drought sensitive cultivar Condor. According to Zang et al. (2018), the expression of *TaPEPKR2* mRNA is induced by heat and 20% PEG treatment in wheat. Moreover, wheat plants overexpressing *TaPEPKR2* present an enhanced response to heat and drought treatments [32]. Protein kinases regulate key aspects of cellular function, including responses to external signals [32,33].

Further, our data showed that the expression level of P5CS in tolerant cultivar (Wadielniel) was significantly higher, as compared to Condor. On the other hand, the expression level was significantly reduced when under drought stress conditions. Interestingly, we noticed that the induction of higher P5CS expression preceded the accumulation of proline under drought stress. Similarly, P5CS gene upregulation under drought stress has also been observed in wheat [34,35,36,37]. It is clear that plants overexpressing P5CS accumulate more proline than the control plants and are tolerant to osmotic stress [38]. Here, we only highlighted a few genes that might regulate the responses to drought stress in wheat, but further experimental work is needed to assess their roles in improving drought tolerance.

### 3.3. Drought Stress Produces Drastic Metabolic Changes

Metabolites play a major role in drought tolerance, as some of them have been considered as signaling molecules [10]. GC–MS is one of the major analytical tools in metabolomics to detect, identify, and analyze small molecules. In this study, we used metabolomic analysis to study the response of wheat cultivars to drought stress. A total of 42 metabolites in Condor and 26 in Wadielniel were significantly changed under drought stress conditions. The most significant changes occurred for amino acids, organic acids, and sugars. Proline, 4-hydroxy proline, valine, and glutamine, which are known to protect plants against abiotic stress, were more abundant in the drought treatments. Similarly, increased levels of amino acids were recorded under drought stress in other wheat studies [11,12,39]. Arginine, histidine, lysine, glycine, and β-alanine occurred at higher abundances in the drought sensitive cultivar Condor. The increase in these amino acids probably resulted from enhanced stress-induced protein breakdown or the inhibition of protein biosynthesis [40].

The most significantly changed metabolite was proline. Dramatic increases of proline levels under water-deficit conditions were previously found in wheat and maize cultivars of differing drought tolerance [41]. Proline acts as an osmolyte for osmotic adjustment and contributes to stabilizing sub-cellular structures, preventing oxidative burst, and accounting for higher drought tolerance [42].

Organic acids play an important role in energy production. They are precursors of amino acids, and may modulate plant adaptation to stress, including drought [43]. The current study shows that Wadielniel, the drought tolerant cultivar, accumulates high levels of glycolic acid, malic acid, fumaric acid, and pipecolic acid; these organic acids have important roles in the response to drought in wheat, and their levels can be related to drought tolerance. Kang et al. (2019) found that the accumulation of some organic acids, including glycolic acid, could contribute to greater capacity of some genotypes of wheat to manage drought stress [44]. For example, Fernie and Martinoia (2009) stated that besides being an essential storage carbon molecule during drought, malate has also a notable role in pH balancing and stomatal function [45]. Besides organic acids, some sugars showed high content upon drought. Sucrose had a higher content in the drought tolerant cultivar. A previous study has shown a high amount of sucrose in a drought-tolerant wheat genotype under drought stress [11]; it has been demonstrated that the presence of monosaccharides enables plants to stimulate efficient defense mechanisms [46,47]. Quinic acid as well as *myo*-instol were significantly reduced under drought stress in both cultivars. Correia et al. (2018) and Obata et al. (2015), unlike what was observed under drought stress in this study, found that heat and drought stress activated the shikimic acid pathway, and increased *myo*-inositol levels in maize and *Eucalyptus globulus*, respectively [10,48].

Another response to different stresses is the change in the composition of membrane lipids, which is highly variable depending on the stress and the species. We observed a general decrease in phosphatidylcholine (PC) and phosphatidylethanolamine (PE) on both cultivars subjected to drought stress. This pattern is similar in olive tree [49] and in wheat seedlings [50] treated with polyethylene glycol (PEG), which mimics the drought stress, that also present a decrease in PC and PE content upon drought stress. PC is the main substrate of phospholipase D α (PLD) and alteration on its expression would change the membrane composition. Arabidopsis plants overexpressing wheat PLDα present a better response to drought than the wild-type plants [51]. Phospholipids as principal components of the cell membrane play a vital role for maintaining its stability. A decrease on PC and PE will result in an unstable membrane with an increased ion permeability [52]. We also observed a higher concentration of digalactosyl-diacylglycerol (DGCG), similar to that found in maize [53], as well as in wheat seedlings [50]. This higher concentration of DGCG is a common strategy in plants to preserve membrane stability [54]. The results presented here provided a list of metabolites that play significant roles to enhance drought tolerance in wheat cultivars, and could be potential biomarkers for drought-stress responses.

## 4. Conclusions

Collectively, our results suggest that the selected drought-tolerant wheat cultivar Wadielniel has a greater capacity in regulating water deficit stress than the drought-sensitive cultivar Condor. As suggested by an enhanced physiological response supported by upregulating regulatory genes and producing more sugars, organic acids and important amino acids in shoots, which enable the plant to maintain growth under water deficit stress conditions. Those results enhance the knowledge on wheat responses to this stress, which cause severe yield loses and might be used as potential crop improvement in breeding programs.

## 5. Materials and Methods

### 5.1. Plant Material and Water Deficit Stress Experiment

Two wheat (*Triticum aestivum* L.) cultivars with contrasting drought response were used in this work. Wadielniel is an Egyptian cultivar (Giza 160) and presents great drought resistance, while Condor is less resistant. Both cultivars are extensively used in Sudan since 1987 and 1979, respectively [55]. Five plants per genotype at DAS 22 (T0) and five per genotype and treatment at T2 (DAS 59) were phenotyped in an experiment on a precision phenotyping system and used for genetic and metabolic analysis.

Plants were grown on the phenotyping platform (LemnaTec-Scanalyzer 3D system; LemnaTec GmbH, Aachen, Germany) located in a controlled greenhouse at IPK Gaters-leben (51°49′23″ N, 11°17′13″ E, 112 m a.s.l.). Plants were transported automatically to im-aging chambers equipped with top and side view RGB and fluorescence cameras, while a balance-watering station enabled controlled watering [56]. Each pot was filled in a standardized manner with Klaßman number two substrate [57] and with 7 g fertilizer (19%N, 9%P_2_O_5_ and 10%K_2_O), and placed on the platform. Two seeds per pot were sown, and seedlings thinned out to one plant per pot at DAS 7. Plants were subjected to drought reducing the watering level to 20% (PAW) content, 23 days after sowing (DAS). This level was selected based on previous studies in barley, where this water content stopped barley growth [18]. Control plants were maintained at 70% PAW and plants were watered and imaged daily from side and top view. Greenhouse lights were set to a day length of 15 h, while temperature was changed along the experiment growth period: (i) from sowing until 22 DAS (T0) 18/16 °C day/night (ii) from 23 DAS to 61 DAS 20/16 °C and (iii) from 62 DAS to maturity at 24/20 °C. Pots were automatically randomized by modi included in the software, several times a week.

**Figure 8:**
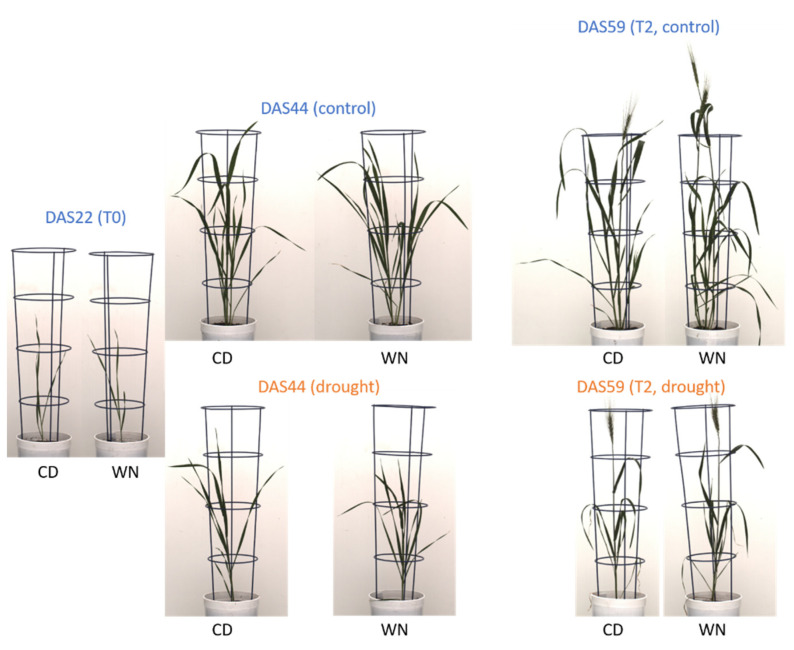
Individual plant images from RGB side view camera for plant at harvesting times T0 (DAS 22), DAS 44, and T2 (DAS 59) from Condor (CD) and Wadielniel (WN).

Samples used for metabolomics and gene expression were harvested at DAS 22 (T0) as a control, as well as DAS 59 (T2) when the PAW had reduced in all plants to a stable level of 20% and all plants had reached the flowering stage. Samples were frozen in liquid nitrogen and stored at −80 °C until further use. At T0, the youngest fully developed leaf was harvested, at T2 the flag leaf from each main tiller and the leaf below were taken. An impression of the different growth response from both genotypes is presented in Figure 8.

Using these photography’s RGB side view images, we determined the day of heading (BBCH 55, half of the ear is out of the flag leaf) [17], the number of tillers, as well as the number of ears at DAS 71, 85, and 98. The number of tiller was counted manually before and during drought stress at DAS 22 (T0), 44 and 53. After flowering, the number of ears was determined by visual inspection of RGB side view images at DAS 71, 85, and 98. At maturity, we determined plant height excluding awns, the length of ear and awns (of the main ear) the length of peduncle, and the length of the last internode. We determined the weight of total above ground biomass, then threshed the plant and evaluated the grain weight, then calculated the harvest index and the straw weight. Further, we determined the total number of seeds (whole plant and main ear) and using the Seed Analyzer “Marvin” (GTA Sensorik GmbH, Neubrandenburg, Germany), we measured the Thousand kernel weight (TKW), average seed area, length, and width. The number of spikelets of the main ear were also counted and we calculated the number of seeds per spikelet.

### 5.2. Gene Expression Analysis

Total RNA was extracted using Trizol reagent (Life Technologies) following manufacturer instruction, and quantified using a Nano-Drop 8000 spectrophotometer. cDNA was synthesized from 300 ng RNA using Superscript III reverse transcriptase (Invitrogen, http://www.invitrogen.com/, accessed on 8 December 2021), with oligo (dT) primers, dNTPs (10 mm) and RT buffer, and incubated at 65 °C for 5 min. After Superscript III reverse transcriptase, Superscript III buffer and DTT and were added, and the mixture was incubated at 42 °C for 1 h, followed by 10 min at 70 °C. Using SYBR Green (TaqMan) fluorescent dye, qRT-PCR reaction were performed as described in [58]. PCR was performed using an ABI PRISM 7900HT sequence detection system (Applied Biosystems), using at least five biological replicates and the *TaACTIN* gene as control. The qPCR reactions were followed the recommended thermal profile: 95 °C for 10 min, then 40 cycles of 95 °C for 15 s, 60 °C for 1 min, and 72 °C for 30 s, and a final cycle of rapid heating to 95 °C to denature the DNA, followed by cooling to 55 °C for the melting curve. The relative levels of RNA for each gene were calculated from cycle threshold values according to the 40-ΔCt method according to [59]. The primers used and gene numbers are listed in Table S2.

### 5.3. Metabolite Profiling

Extraction of metabolites was performed in accordance with [60]. Briefly, 100 mg of fresh material was extracted with 1 mL of methyl-*tert*-butyl-ester:methanol (3:1), followed by a phase separation adding H_2_O:MeOH (3:1 *v/v*). For lipids analysis, 250 µL from the aqueous phase were taken and dried under vacuum. The dried residue was resuspended in 100 µL of UPLC-grade acetonitrile:isopropanol (70:30) mixture. From that, 2 µL were injected individually on Acquity UPLC system using an RP C8 column and analyzed by MS [61]. The samples were measured in positive and negative ionization mode. The mass spectra were acquired using an Orbitrap high-resolution mass spectrometer: Fourier-transform mass spectrometer (FT-MS) coupled with a linear ion trap (LTQ) Orbitrap XL (ThermoFisher Scientific, Waltham, MA, USA, https://www.thermofisher.com, accessed on 8 December 2021). On the other hand, 100 µL from the organic phase were dried for primary metabolite analysis. The polar fraction was dried under vacuum, and the residue was derivatized for 120 min at 37 °C (in 40 μL of 20 mg mL^−L^ methoxyamine hydrochloride in pyridine), followed by a 30 min treatment at 37 °C with 70 μL of MSTFA [62]. An autosampler Gerstel Multi-Purpose system (Gerstel GmbH & Co.KG, Mülheim an der Ruhr, Germany) was used to inject the samples to a chromatograph coupled to a time-of-flight mass spectrometer (GC-MS) system (Leco Pegasus HT TOF-MS (LECO Corporation, St. Joseph, MI, USA). Helium was used as carrier gas at a constant flow rate of 2 mL/s, and gas chromatography was performed on a 30 m DB-35 column. The injection temperature was 230 °C and the transfer line and ion source were set to 250 °C. The initial temperature of the oven (85 °C) increased at a rate of 15 °C/min up to a final temperature of 360 °C. After a solvent delay of 180 s, mass spectra were recorded at 20 scans s^−1^ with *m*/*z* 70–600 scanning range. Chromatograms and mass spectra were evaluated by using Chroma TOF 4.5 (Leco) and TagFinder 4.2 software.

Metabolite data correlation was analyzed using the website MetaboAnalyst [63] and Expressionist Analyst 14.0.5 (Genedata, Basel, Switzerland) (https://www.genedata.com/products/expressionist, accessed on 8 December 2021). Univariate analysis (two paired *t*-test) was applied to calculate the statistical significance and fold change of the metabolites between two time points (stress over control). The supervised multivariate method, PLS-DA (partial least squares-discriminant analysis) was used to maximize the metabolome difference between the control and stress treated samples, as well as the difference between two cultivars. The relative importance of each metabolite to the PLS-DA model was checked using variable importance in projection (VIP). Metabolites with VIP >1.0 were considered as differential metabolites for group discrimination. Heat maps were generated based on log_2_-transformed fold change values.

### 5.4. Statistical Analysis

Statistical significance was tested with a Student’s *t*-test using Graphpad 9.0. Differences were calculated either between conditions in the same cultivar or between cultivars in different conditions, and are stated in each figure or table.

## Figures and Tables

**Table 1:** Yield related characteristics presented as mean ± SD. Statistical significances are determined by a Student *t*-test between cultivars in the same experimental conditions.* *p* < 0.05, **** *p* < 0.0001.

Genotype		Spikelet Number	Spikelet with Seeds	Seeds per Spikelet	Total Plant Grain Number	Grain Number of Main Ear
Wadielniel	Control	19.63 ± 1.82 *	17.56 ± 2.39	1.79 ± 0.54	219.85 ± 40.87	35.31 ± 11.22
	Drought	18.67 ± 1.67 ****	16.82 ± 2.23 ****	1.51 ± 0.35	30.42 ± 9.47	28.17 ± 7.20
Condor	Control	17.44 ± 1.76	16.50 ± 2.31	2.26 ± 0.41 *	217.15 ± 25.07	40.67 ± 9.48
	Drought	15.15 ± 0.9	13.38 ± 0.96	1.87 ± 0.18 *	28.38 ± 3.78	28.38 ± 3.78

**Table 2:** Seed parameters presented as means ± SD. Statistical significances determined by a Student *t*-test between cultivars in the same experimental condition. * *p* < 0.05, ** *p* < 0.01, *** *p* < 0.001.

Genotype		TKW (g)	Seed Area (mm^2^)	Seed Width (mm)	Seed Length (mm)
Wadielniel	Control	46.15 ± 4.94 **	15.88 ± 1.03 **	3.55 ± 0.15 *	6.24 ± 0.20 ***
	Drought	35.55 ± 8.24 *	13.39 ± 1.58 *	3.18 ± 0.23	6.02 ± 0.24 *
Condor	Control	40.32 ± 5.11	14.45 ± 1.10	3.38 ± 0.21	5.94 ± 0.16
	Drought	30.72 ± 3.09	12.41 ± 0.73	2.98 ± 0.14	5.78 ± 0.17

## Data Availability

All the data reported are found either in the manuscript or in the Supplementary Material  of the article.

## Supplementary Materials

The following are available online at https://www.mdpi.com/article/10.3390/ijms222413287/s1.
