# Supplementary material for: Comparative Molecular and Metabolic Profiling of Two Contrasting Wheat Cultivars under Drought Stress
Source: Int J Mol Sci. 2021 Dec 10;22(24):13287. doi: 10.3390/ijms222413287 (PMC8707805; doi:10.3390/ijms222413287)
Supplement: Supplementary file 1 [file ijms-22-13287-s001.zip › Figure S1.pdf]

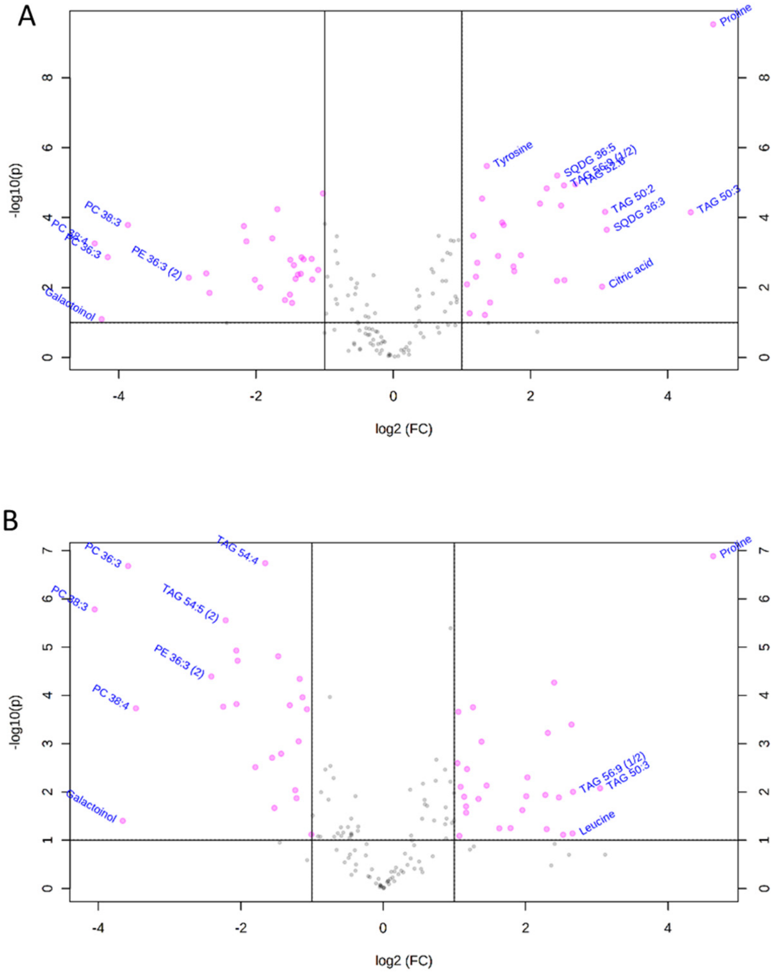


Figure S1: Volcano plots for metabolite profiling. Volcano plots for all metabolites measured by GC-MS or UPLC-MS. (A) Volcano plot data from Condor samples, (B) Volcano plot graph represents data from Wadielniel. Volcano plots showing metabolites with fold changes (FC) at ($p \leq 0.05$), Condor or Wadielniel control samples compared to drought samples for each cultivar. The pink circles and names in blue indicate metabolites with FC either $\geq 2$ or $\leq 0.5$ at a $p \leq 0.05$. Calculations were performed by means of the MetaboAnalyst software
