# Supplementary material for: Comparative Molecular and Metabolic Profiling of Two Contrasting Wheat Cultivars under Drought Stress
Source: Int J Mol Sci. 2021 Dec 10;22(24):13287. doi: 10.3390/ijms222413287 (PMC8707805; doi:10.3390/ijms222413287)
Supplement: Supplementary file 1 [file ijms-22-13287-s001.zip › Table S2.pdf]

Genes names and codes	Forward sequence 5’- 3’	Reverse sequence 5’- 3’
TaActin AB181991.1	CTTGTATGCCAGCGGTCGAACA	CTCATAATCAAGGGCCACGTA
TaPEPKR2-L TraesCS5B02G243700	ATGGAGTCGCTGCCGCGGAAG	ATTGGGGTCATGTCAGAACGCC
TraesCS5A02G411700 JUB1 orthologue from AT	GACACCATGCACAACCAACATTTC	TTGCGGTTTCTGCTCCGGTATC
TraesCS5D02G148800.1 (NAC29) 5D ATAF1 orthologue from AT	ATCCTCCATGAGGCTGGATGAC	AGCTCTCCTGCTCATGCTCTTG
TaD27 TRIAECS427BLTGACv1_577741_AA1882380	ATGGAGGCCACCGCACTTGTGC	CTAGCAATTCACACCATAGTCCT
TaP5CS Traes3DL3E215D878.2	TAAATGCCGTTGTTGCTGCC	AGCAAAACTAACAATGGCTACCAG
TaDREB2 TraesCS5A01G310100.1	GCGTACAACACCTTGATTTCC	AAACTCAACTCACATCTAAGC
TaNAC55A TraesCS3A02G250800.1	GCCCTTGAAAGCCGTAATTGCC	TGCTGTACTGCTGCTGCTTG
TaNAC55B TraesCS2B02G075900	CACGAGTTCAGGTTCCCTTCAC	CTTGTCGATCGGCCTACTCTTG
TaNAC55D TraesCS2D02G061500	AAACACAGCCGCCATCACAGAG	ACTGCGCGTCAAAGAAGATCGG
TaNAC7A TraesCS5A02G411700.1 5A	GACACCATGCACAACCAACATTTC	TTGCGGTTTCTGCTCCGGTATC
TaNAC7B TraesCS5B02G415600	AGTCAGATGGACGCAGTGAACG	AACGGCTGGTTGTGCATGGTATC
TaNAC7D TraesCS5D02G420800	CACCATGCACAACCAGCAGTTTC	TTGTGGTTTCTGCTCCGGCATC

Table S2 List of qPCR Primers used in this study
